# ISCB-Student Council Narratives: Strategical development of the ISCB-Regional Student Groups in 2016

**DOI:** 10.12688/f1000research.10420.1

**Published:** 2016-12-20

**Authors:** Sayane Shome, Pieter Meysman, R. Gonzalo Parra, Alexander Miguel Monzon, Nicolas Palopoli, Benjamen White, Farzana Rahman, Mehedi Hassan, Zeynep Özkeserli, Efejiro Ashano, V. Keith Hughitt, Muhammad Uzair Khan, Denis J. Murphy

**Affiliations:** 1 Bioinformatics and Computational Biology Program,, Iowa State University, Ames, USA; 2 Advanced Database Research and Modeling group (ADReM), Department of Mathematics and Computer Science, University of Antwerp, Antwerp, Belgium; 3 Biomedical Informatics Research Network Antwerp (biomina), University Hospital Antwerp/University of Antwerp, Antwerp, Belgium; 4 Protein Physiology Laboratory, Departamento de Química Biológica, Facultad de Ciencias Exactas y Naturales, UBA-CONICET-IQUIBICEN, Buenos Aires, Argentina; 5 Quantitative and Computational Biology Group, Max Planck Institute for Biophysical Chemistry, Goettingen, Germany; 6 Departamento de Ciencia y Tecnología, Universidad Nacional de Quilmes, CONICET, Buenos Aires, Argentina; 7 Unidad de Físico Química, Departamento de Ciencia y Tecnología, Universidad Nacional de Quilmes, CONICET, Buenos Aires, Argentina; 8 Fundación Instituto Leloir-IIBBA-CONICET, Buenos Aires, Argentina; 9 Earlham Institute, Norwich Research Park, Norwich, UK; 10 Genomics and Computational Biology Research Group, Faculty of Computing, Engineering and Science, University of South Wales, Wales, UK; 11 Medical Biotechnology Program, Bioinformatics Group, Institute of Medical Sciences, Acibadem University, Istanbul, Turkey; 12 Immuno-virology and Vaccine Development Unit, Medical Biotechnology Department, National Biotechnology Development Agency, Abuja, Nigeria; 13 Bioinformatics Research Group, Covenant University, Ota, Nigeria; 14 Department of Cell Biology and Molecular Genetics, University of Maryland, College Park, USA; 15 Center for Bioinformatics and Computational Biology, University of Maryland, College Park, USA; 16 Biotechnology and biotechnology program. Institute of integrative biosciences, CECOS University of Information Technology and Emerging Sciences, KPK, Pakistan

**Keywords:** Student organizations, Bioinformatics, Computational Biology, Workshops, Virtual seminars, ISCB Student Council, Regional Student Groups

## Abstract

Regional Student Groups are groups established and managed by the ISCB-Student Council in different regions of the world. The article highlights some of the initiatives and management lessons from our 'top-performing' Spotlight Regional Student Groups (RSGs), RSG-Argentina and RSG-UK, for the current year (2016). In addition, it details some of the operational hurdles faced by RSGs and possible solutions.

## Introduction

Since its inception in 2004, the ISCB-Student Council Regional Student Group (RSG) program has burgeoned to 29 RSGs, which are actively operating in various parts of the world. RSGs provide a platform to connect young researchers at different tiers of the education system (including undergraduates, masters, PhD and postdocs) within and outside of their own region. They organize various offline ^1^ and online events with the objective to disseminate information and provide training about topics related to computational biology. In addition, these events double as an excellent networking opportunity. While undertaking such initiatives, the organization learns priceless lessons on management, organizational behavior and the significance of knowledge transfer to accelerate scientific research ^2^. Here we highlight some of the initiatives and management lessons from our 'top-performing' Spotlight RSGs of the year 2016. Further, we elaborate some of the key challenges RSGs face and case studies of how they resolved these.

## Spotlight RSG of the year 2016: RSG-Argentina

Since its initiation in 2012, RSG-Argentina has grown steadily, organizing different events, including workshops, local meetings and expanding its regional influence on a continuous learning path full of valuable lessons ^3^.

In the past two years, two workshops were organized as a part of the annual RSG-Argentina Workshop Series. Further, a Wikipedia Hackathon was organized at three different locations in the country. It is worth noting that one of the participating teams was also recognized with the 2nd prize at the ISCB Wikipedia International Competition for their contribution to the BioJava entry in the Spanish language.

In 2016, RSG-Argentina organized its first independent event, the 1st SAJIB (the Argentine Symposium for Young Researchers in Bioinformatics). This event constituted a forum where students and early career researchers got together to discuss their science and strengthen the local scientific network ^4^. The meeting was organized as a two-day event at the Buenos Aires University. The first day had a traditional symposium format with around 50 attendees from different parts of the country, 3 keynote speakers, 7 student talks and 2 poster sessions where 25 posters were exhibited. Two hands-on workshops were offered on the second day. This marked a milestone for RSG-Argentina, organizing its first autonomous national symposium.

When RSG Argentina was taking its first steps, many things ended up being improvised or incompletely executed. Since then, this group has grown to become a well-organized RSG with demonstrated capacity to organize student-based events. The key for this successful growth has been the building of a strong leadership composed of at least 7–9 people who are active at any moment and willing not only to discuss work matters, but also have a fun time together. Moreover, the strong presence and communication with their community by mailing lists, social networks and at different events has created a solid base of members that regularly interact with each other. The pro-active dynamic member community has allowed RSG-Argentina leaders to get involved in regional events as well. Both Latin American SCS versions have had RSG-Argentina leaders as symposium chairs.

## Spotlight RSG of the year 2016: RSG-UK

Inspired by the success stories of existing RSGs, RSG-UK was formed in December 2013 with a vision to strengthen and enhance the networking potential among young computational biologists and bioinformaticians working and studying in the UK. In October 2014, RSG UK organized its inaugural scientific event “1st Student Symposium on Computational Biology and Life Sciences” at the University of South Wales ^5^. The first initiative received wide accolade where delegates from 24 British institutions and industries attended and presented cutting-edge research. The event was streamed live and participants from 8 other European RSGs actively contributed in the online discussions on various social platforms. The talks from the session were uploaded to YouTube for prosperity.

In continuation with the success of the previous events, RSG-UK and the High-Performance Computing Wales (HPCW) consortium co-organized a supercomputing workshop targeting advanced level biology undergraduates on how to answer biological questions using supercomputing clusters. The event was co-supported by Fujitsu Laboratories Europe Ltd. and the University of South Wales. The workshop helped the attendees to understand the robustness of the computational biology field and the areas they can explore as part of their future career or higher education.

In 2015, RSG-UK grew welcoming new members and organized its second symposium in October at the Earlham Institute, Norwich, England (previously known as The Genome Analysis Centre) ^6^. Both RSG-UK symposia covered keynote talks, student presentations and postdoc presentations, career talks from senior and junior principal investigators. These symposia were also sponsored by and had representation from key industrial players, e.g. Illumina, Inc. and Fujitsu Laboratories Europe Ltd., which helped to promote relationships between RSG-UK, its members, and industry.

Since its beginnings, RSG-UK's committee members have actively participated in the organization of the Student Council's major symposia, such as SCS 2014–16, ESCS 2016. The founding members of the team have also developed a bespoke symposium management system that is now widely used to organize Student Council's flagship events (SCS 2015, 2016, European-SCS 2016).

By continuing to connect existing student communities at research institutes across the UK through training events, workshops, and symposia, they aim to build a robust network that supports professional and soft-skill development helping prepare students for a successful scientific career.

## Prime highlights about strategies to overcome operational hitches

The management team of RSGs face various hurdles when organizing their activities, many of which are common to all RSGs. Here we highlight some RSG case stories about different issues that were faced and how they were addressed.

1) Logistics costs and ways to minimize it.

RSG-Turkey chose another way of bringing people together: using online tools. Limited resources in certain countries, such as developing nations, often impede the exposure and access to content that students in countries with more resources cherish. Driven by this motivation, the BioInfoNet webinar program was established in 2013. The program’s goal is to conduct online seminar series open to anyone around the world as a journal club, scientific session, career-related discussion, and more. So far, BioInfoNet has hosted eight webinars (as of August 2016) on the BigMarker platform, and these webinars have had more than 2000 views in total, indicating the widespread and international success of the program.

Even though webinars provide a very good opportunity to participate in scientific meetings, RSG-Turkey still values symposia as a means of improving social interactions between group members. The latest student symposium took place on October 17th, 2015, at Muğla, Turkey. One of the key highlights of the symposium was the “Science Slam: 5 slides under 5 minutes” session, during which, the students transmitted the general overview of their research project to the audience using at most 5 slides and under 5 mins (3–4 mins presentation, 1–2 mins discussion). In total, 7 students shared their projects and the interaction between the students made it clear that it should be a-must-have-session in the following meetings. Interactive activities such as ice breaking and science slam are therefore strongly recommended to RSGs planning to organize such activities during student meetings. Due to the scarcity of the funds, the keynote lecture by Dr. Jörg Menche from CEMM, Austria was delivered as a webinar.

2) Interdisciplinary nature of the field and how to set the target audience for the sessions.

On June 15, 2016, the Washington DC area RSG (RSG-DC) held its first-ever workshop on bioinformatics and computational biology at the University of Maryland, College Park campus. The single-day event featured six 1.5 hour workshop tutorials presented by students and postdocs. The event was a huge success with nearly 100 attendees from over twenty different institutions in the area. Attendees ranged in experience from high school students to senior investigators, with graduate students forming the largest subgroup. Interestingly, many of the attendees were not trained bioinformaticians/computational biologists, but rather came from a diverse set of fields including molecular biology, ecology, biomedicine and plant biology. This presented a challenge with respect to the target level of the workshop talks. This was resolved by providing low-level and accessible tutorials, rather than assuming a large amount of background knowledge that many of attendees would, likely, be lacking. In the end, this strategy seems to have worked well, and although none of the attendees are likely to have left as experts in the workshop topics, they obtained basic insights in whether specific tools and techniques could be useful in their own research.

Apart from workshop proceedings, one of the primary goals of the meeting was to bring together students and other researchers from the DC region and provide them with a chance to meet one another and exchange ideas. This objective was served by including several scheduled breaks throughout the day where participants had the chance to chat with one another over food or coffee. By surveying attendees before the meeting, they were able to group them into one of several “clusters” based on the similarity of their research interests and backgrounds. Color-coded stickers were added to the attendee name badges to help make it easier to spot others with similar interests. Based on feedback following the event, participants largely found the workshop to be helpful and appreciated the chance to connect with other researchers from outside of their home institutions. The workshop organizers also learned a lot from the experience, and hope to plan similar events in future.

3) Managing transition plans for sustenance of the RSG in the region.

After starting up in 2010, RSG-Pakistan encountered a stage of dormancy in 2012. The team encountered various challenges including a re-shifting of base to Peshawar and establishing a new leadership at the location where bioinformatics is not a common subject. Indeed, only one university offers a degree in computational biology in Peshawar. RSG-Pakistan decided to expand its team and also strengthened their presence in the region by conducting symposia related to the field. In order to gather more workforce dedicated to the work, they organized rigorous recruitment drives as well as in-person meetings to create cognizance about the initiative. One of the prime objectives RSG-Pakistan propagated includes awareness about the importance of programming for a conventional biologist, which life sciences curricula generally lack. To this end, they organized ‘Hour of Code’, an event targeted towards undergraduates from the field of biotechnology. After a successful event at the public university, RSG-Pakistan preferred to change the venue to a private university. The new private university campus was more welcoming and open to conduct the events, with significant media coverage via local newspapers on RSG-Pakistan. The recruitment drives, awareness sessions, and spread of word have helped to establish the new team. In addition, establishing the base of operations in a university/research institution, where the organizing team can avail resources easily, always helps.

4) Demographic and linguistic barriers.

RSG Western Africa (WA) comprises of 18 countries out of which 4 actively participate: Ghana, Mali, Niger and Nigeria. In 2014 and 2015 respectively, the RSG successfully held two programs targeted at postgraduate students and early researchers in these countries. Including co-hosting a 5-week H3AbioNet workshop in bioinformatics with the Covenant University Bioinformatics Research Group. The other event was a 6-days “R Clinic” workshop in R programming. Both were held in Nigeria, having the highest number of members and also being the RSG’s operational base.

It has been noticed that institutions with large bioinformatics and computational biology components, such as H3AbioNet/H3Africa, in these areas play a prominent role in the involvement of specific countries. Another factor affecting these demographics is language. West African countries are either Anglophone or Francophone, with a larger number of countries constituted of the latter. Most of the events have been organized in English which may have affected representation from the French-speaking countries.

RSG-WA attempted to address this challenge and attract its Francophone counterparts by organizing a French version of the event and hiring a translator for events. Both of which significantly raised the event budgets and were abandoned after some time. The RSG is planning another event in early 2017 and intends to include Benin (Francophone) on its list of actively participating countries taking advantage of growing collaborations with institutions in that country.

5) Promoting collaborations within and amongst RSGs.

As one of the objectives of the RSG program, we have observed various collaborations within RSGs that are located in the same region, as well as between different regions. Previously RSG-Belgium, Netherlands, Luxembourg and France have collaboratively organized symposiums under the BeNeLuxFr acronym.

Similarly, in the past few years we have witnessed the rise of various RSGs in the Latin American (LA) region. First, RSG-Brazil was created in LA, in 2010, but unfortunately it went inactive after some time. In 2012, RSG-Argentina was officially recognized. Since then, and taking impulse every two years with the ISCB-LA conference, more RSGs have emerged in the region. Following the re-activation of RSG-Brazil in 2013, RSGs in Chile, Mexico and Colombia have come into existence in the following years. Moreover, students from countries without RSGs, are coming forward to participate. Increasing representation of Latin America in the computational biology student community and fostering stronger bonds in the region.

Given the revitalized activity in LA, supported by the emergence of several new leaders in different countries, who started meeting regularly in the annual ISMB conference, a Latin American version of the traditional Student Council Symposium was launched. The 1st LA-SCS 2014 was held in Belo Horizonte, Brazil ^7^, followed by the recent 2nd LA-SCS 2016 in Buenos Aires, Argentina. In parallel to an increasing international networking among students, RSGs on their own are getting bigger and more dynamic. The challenge now is not only to maintain the initiatives in the current RSGs, but to try to create new ones and extend the type of activities organized by pre-existing RSGs to the newly created RSGs. With intention to sustain a dynamic development of LA RSGs, accompanied by a secured turnover of the leadership. Great things are still to come for this continent.
